# Tumor-Intrinsic Mechanisms Regulating Immune Exclusion in Liver Cancers

**DOI:** 10.3389/fimmu.2021.642958

**Published:** 2021-04-26

**Authors:** Katherine E. Lindblad, Marina Ruiz de Galarreta, Amaia Lujambio

**Affiliations:** ^1^Department of Oncological Sciences, Icahn School of Medicine at Mount Sinai, New York, NY, United States; ^2^Liver Cancer Program, Division of Liver Diseases, Department of Medicine, Icahn School of Medicine at Mount Sinai, Tisch Cancer Institute, New York, NY, United States; ^3^Icahn School of Medicine at Mount Sinai, The Precision Immunology Institute, New York, NY, United States; ^4^Graduate School of Biomedical Sciences at Icahn School of Medicine at Mount Sinai, New York, NY, United States

**Keywords:** hepatocellular carcinoma, immune escape, immune exclusion, immunotherapies, CTNNB1

## Abstract

Representing the fourth leading cause of cancer-related mortality worldwide, liver cancers constitute a major global health concern. Hepatocellular carcinoma (HCC), the most frequent type of liver cancer, is associated with dismal survival outcomes and has traditionally had few treatment options available. In fact, up until 2017, treatment options for advanced HCC were restricted to broad acting tyrosine kinase inhibitors, including Sorafenib, which has been the standard of care for over a decade. Since 2017, a multitude of mono- and combination immunotherapies that include pembrolizumab, nivolumab, ipilumumab, atezolizumab, and bevacizumab have been FDA-approved for the treatment of advanced HCC with unprecedented response rates ranging from 20 to 30% of patients. However, this also means that ~70% of patients do not respond to this treatment and currently very little is known regarding mechanisms of action of these immunotherapies as well as predictors of response to facilitate patient stratification. With the recent success of immunotherapies in HCC, there is a pressing need to understand mechanisms of tumor immune evasion and resistance to these immunotherapies in order to identify biomarkers of resistance or response. This will enable better patient stratification as well as the rational design of combination immunotherapies to restore sensitivity in resistant patients. The aim of this review is to summarize the current knowledge to date of tumor-intrinsic mechanisms of immune escape in liver cancer, specifically in the context of HCC.

## Introduction

### Liver Cancer and Hepatocellular Carcinoma

Liver cancers represent the fourth leading cause of cancer-related mortality worldwide, with estimates from the World Health Organization predicting over 1 million deaths in 2030 (1). As the second-most lethal malignancy behind pancreatic cancer and harboring a 5-year survival rate of 18%, liver cancers represent a major global health concern (1). The two most frequent forms of liver cancer are hepatocellular carcinomas (HCCs) and cholangiocarcinoma, which represent 80–90% and 6–15% of all primary liver cancers, respectively (2). While patients diagnosed with early-stage HCC may be eligible for potentially curative surgical resection, most patients are diagnosed with recurrent or advanced stage disease (1). Until recently, treatment options for advanced HCC were restricted to tyrosine kinase inhibitors (TKIs) that confer limited survival benefits (3–6). Sorafenib, a TKI, has been the standard of care for advanced HCC for over a decade (3) but confers a survival benefit of merely 2.8 months over placebo. More recently, additional TKIs have been approved as first or second-line treatment for advanced HCC patients including regorafenib (4), cabozantinib (5), and ramucirumab (6). However, since 2017, two immune checkpoint inhibitors targeting the programmed cell death-1 (PD-1) pathway, pembrolizumab and nivolumab, the latter alone or in combination with the monoclonal antibody targeting cytotoxic T-lymphocyte-associated protein 4 (anti-CTLA-4) have been FDA-approved as second-line treatment for advanced HCC (7–9). Most recently, the combination of monoclonal antibodies atezolizumab (anti-programmed death ligand-1; anti-PD-L1) and bevacizumab (anti-vascular endothelial growth factor; anti-VEGF) has shown, for the first time in any HCC clinical trial, superiority over sorafenib and is now FDA-approved as first-line treatment for advanced HCC (10). With objective response rates of around 30%, these immunotherapies have demonstrated unprecedented results in the treatment of advanced HCC (7–10). However, this also means around 70% of patients are insensitive to this treatment, making it imperative to understand mechanisms of immune escape in liver cancers in order to design novel combination therapies that restore sensitivity in these immunotherapy-resistant patients as well as identify biomarkers of resistance or response to improve patient selection.

### Immunoediting and Immune Escape

To understand the notion of immune escape in cancer, it is important to first understand the concept of cancer immunoediting as well as the cancer immunity cycle. Immunoediting describes the process by which the immune system protects the host from cancers (i.e., immune surveillance); however, in doing so the immune system also places evolutionary pressure on malignant cells causing them to undergo immunogenic sculpting that enables disease progression (i.e., immune escape) (11, 12). Immunoediting proceeds through three phases: elimination, equilibrium, and escape (11). During the elimination phase, transformed cells that have escaped normal cell-intrinsic apoptotic/senescence checkpoints are recognized and killed by cells of the innate and adaptive immune systems (11). In the equilibrium stage, tumor subclones that survived the elimination phase (e.g., through the acquisition of additional genetic alterations that promote immune suppression) begin to expand (11). However, the overall net growth of the tumor is still being prevented primarily by the adaptive immune system, which maintains tumor cells in a state of functional dormancy (11, 12). Over time, the evolutionary pressure placed on the developing tumor by the immune system, coupled with the genetic instability associated with rapidly dividing malignant cells, promotes the selection and expansion of tumor subclones that have acquired alterations that suppress host immune responses and tumor cell destruction (11, 12). In this final stage of escape, tumor outgrowth is no longer restricted or blocked by the host immune responses and these tumor subclones emerge to cause clinically apparent disease (11, 12). While the immune system is capable of recognizing and killing malignant cells and constraining tumor growth, this theory of cancer immunoediting describes the process by which the same mechanism also promotes the emergence of malignant subclones that have undergone immunogenic sculpting to evade detection and destruction.

**Figure 1:**
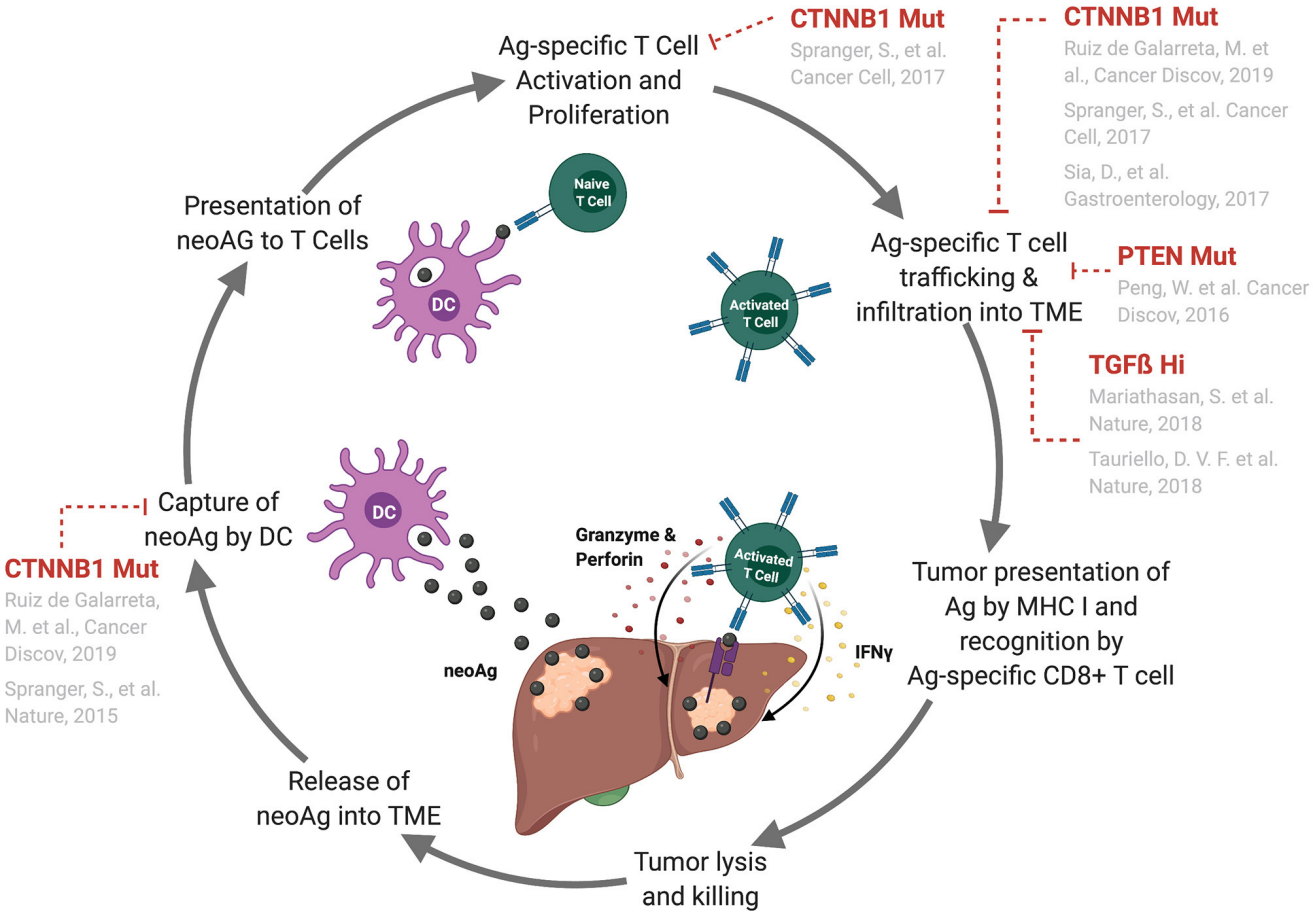
Genetic alterations that perturb the cancer–immunity cycle and lead to tumor immune escape. Depiction of the cancer-immunity cycle (13). Tumor cells release antigens into the tumor microenvironment where they are sampled by circulating antigen presenting cells (APCs), such as dendritic cells. These APCs traffic to the lymphoid organs where they present antigens to T cells leading to T cell priming and activation. These activated T cells traffic back to the tumor site where they infiltrate, recognize, and kill tumor cells expressing their cognate antigen. Known mechanisms of immune escape in HCC and other tumor types that perturb specific points in this process are indicated. Abbreviations are as follows: neoAg, neoantigen; TME, tumor microenvironment; DC, dendritic cell; Ag, antigen.

In the cancer immunity cycle (13) (Figure 1), certain somatic mutations in tumor cells result in the production of a modified protein product (neoantigen), which has the potential to be recognized by the host's immune system as foreign. Additionally, cancer-specific antigens resulting from expression of viral genes or aberrant expression can also be recognized by the immune system. These antigens can be released into the tumor microenvironment and sampled by dendritic cells, which travel to secondary lymphoid organs where they prime tumor antigen-specific adaptive (T and B lymphocyte-mediated) immune responses (13). Primed antigen-specific cytotoxic CD8+ T cells subsequently traffic to and lyse tumor cells that are presenting tumor-specific antigens through the Major Histocompatibility Complex (MHC) class I molecules, which results in the release of more tumor-associated antigens into the microenvironment (13) (Figure 1). The cancer immunity cycle represents the adaptive arm of the immune surveillance cancer immunoediting phase. However, innate immune cells, such as natural killer cells and γδT cells, also participate in immune surveillance by these cells (14–16). Tumors can escape immune surveillance through a variety of strategies, such as the acquisition of genetic alterations that perturb the aforementioned processes.

## Tumor-Intrinsic Mechanisms of Immune Escape: Genetic Alterations in Liver Cancer

Known tumor-intrinsic mechanisms that induce immune escape in the context of liver cancer are limited; however, a few studies have demonstrated that mutations affecting the WNT/ß-catenin pathway, which affects ~27–37% of human HCC patients, promote immune escape in HCC. One group published correlative data on human HCC samples suggesting that ß-catenin activation may promote immune escape (17). Here, Sia et al. analyzed gene expression profiles from 956 HCC patient samples and virtuallyand, using a non-negative matrix factorization algorithm, separated the gene expression profiles from tumor, stromal, and immune cell compartments. Expression patterns were correlated to immune cell infiltration by pathology and immunohistochemical analysis. Then, using data from The Cancer Genome Atlas (TCGA), these immune cell gene expression profiles were correlated with chromosomal aberrations and mutations. Through this analysis, they found that around 25% of HCCs displayed high expression of inflammatory markers (termed “immune class”), either indicative of an adaptive T cell immune response or an immune exhausted phenotype. However, they also found that tumors with a “*CTNNB1*-mutation gene signature” were specifically excluded from the “immune class.” This CTNNB1 class also showed lower T cell enrichment scores and downregulation of CCL-4. Previously, using an autochthonous mouse model of melanoma, Spranger et al. demonstrated that activation of the ß-catenin pathway led to impaired T cell priming through repression of CCL-4-mediated dendritic cell recruitment to the tumor microenvironment and, subsequently, led to resistance to anti-PD-L1 and anti-CTLA-4 immunotherapy (18). However, the resistance to immunotherapy here could be due to the lack of endogenously activated T cell responses and thus no baseline infiltration into the tumors. A subsequent study by Spranger et al. used adoptive T cell transfer to address this and found that still, adoptively transferred effector T cells failed to traffic to the tumor site and this was due to the absence of CXCL9/10 production from CD103+ dendritic cells (those with the ability to cross-present extracellular antigen to CD8+ T cells) (19).

More recently, using a murine model of HCC based on hydrodynamic tail vein injection of genetic elements *in vivo*, our laboratory demonstrated that activating mutations in *CTNNB1* lead to immune escape in HCC (1). We generated two models with *MYC* overexpression and knockdown of *TP53*; one version that is non-immunogenic (*MYC-luc;sg-p53*) and one that is immunogenic due to expression of 3 model antigens (*MYC-lucOS;sg-p53*). The *MYC-lucOS;sg-p53* model had significantly better overall survival and decreased tumor burden compared with *MYC-luc;sg-p53* mice and this was found to be due to CD8+ T cell-mediated tumor clearance. We then generated two additional models overexpressing an activating form of ß-catenin (encoded by *CTNNB1*): non-immunogenic *MYC-luc;CTNNB1* and immunogenic *MYC-lucOS;CTNNB1*. There was no difference in survival or tumor burden between the non-immunogenic and immunogenic mice expressing an activating form of ß-catenin, which suggests that ß-catenin induces immune escape in the context of antigen expression. We found that this was due to diminished CCL5 expression, which in turn impaired dendritic cell recruitment to the tumor microenvironment and thus led to ineffective CD8+ T cell tumor clearance (1). Furthermore, overexpression of CCL5 restored dendritic cell infiltration into the tumors leading to active immunesurveillance and restored survival in the *MYC-lucOS;CTNNB1* mice. Finally, using TCGA data, Luke et al. showed across multiple tumor types, including HCC, an inverse correlation between a T-cell inflamed gene signature and ß-catenin pathway activation (20). ß-catenin pathway activation was inferred through somatic mutations in pathway signaling elements, pathway prediction from RNA-sequencing data, as well as ß-catenin protein levels (20).

Correlative data from human HCC patients as well as mechanistic studies in mouse models of HCC are highly suggestive that tumor-intrinsic activating mutations in the WNT/ß-catenin pathway promote immune escape and resistance to immunotherapy in HCC. More specifically, these studies suggest the mechanism of immune escape is through defective recruitment of dendritic cells to the tumor microenvironment leading to inferior anti-tumor T cell responses (Figure 1). While mutations affecting the WNT/β-catenin pathway account for a large proportion of human HCC cases (27% to 37%), this disease is highly heterogenous with complex genetic etiology underlying each case. It is unlikely that the mutations affecting the WNT/β-catenin pathway are the sole genetic alterations that perturb effective anti-tumor CD8+ T cell responses and promote immune escape and resistance to immunotherapies in HCC. As immunotherapies have only recently demonstrated success in the treatment of HCC, to date, the roles of additional genetic alterations in promoting immune escape and response or resistance to immunotherapies in the context of HCC have not been well elucidated. In other solid tumor types, for example non-small-cell lung cancer, tumor mutational burden and microsatellite instability/mismatch repair deficiency have been implicated as good predictors of response to immune checkpoint therapies - the idea being that these tumors have a higher probability of expressing immunogenic neoantigens capable of eliciting anti-tumor immune responses (21–24). Currently, there is little evidence suggesting a prominent role of tumor mutational burden and microsatellite instability/mismatch repair deficiency as biomarkers of HCC responsiveness to immunotherapies. Two recent studies suggest that these features are, in fact, infrequent in HCC and poor predictors of response to immunotherapy in HCC (25, 26). As a relatively new field, more studies with larger cohorts of patients are needed to investigate the role of tumor mutational burden and microsatellite instability/mismatch repair deficiency as biomarkers of immunotherapy responsiveness in HCC. However, there are a few other genetic alterations with known relevance to human HCCs that have been shown to mediate immune escape and resistance to immunotherapies in other tumor types.

## Tumor-Intrinsic Mechanisms of Immune Escape: Genetic Alterations in Other Tumor Types

While studies investigating tumor-intrinsic mechanisms of immune escape involving acquisition of genetic alterations in liver cancers, and HCC in particular, are scarce, there are further examples published in the context of other solid tumors that may have relevance in HCC. One study by Peng et al. demonstrated that PTEN loss leads to decreased T cell trafficking to tumors and impaired T cell-mediated tumor killing in a murine model of melanoma (27) (Figure 1). Specifically, PTEN loss induced upregulation of CCL2 and VEGF expression and inhibited tumor cell autophagy (27). In melanoma patients, PTEN loss was associated with lower T cell infiltration in tumors and poorer response to anti-PD-1 immunotherapy (27). Though nothing has been published to date implicating PTEN in promoting immune escape in HCC, PTEN is altered in 7% of human HCC patients (28) making this pathway an appealing option for targeted therapies; however, future studies are needed to demonstrate whether or not tumor-intrinsic loss of PTEN leads to immune escape in HCC.

Another example of a tumor intrinsic mechanism of immune escape in cancer is overexpression of the Notch signaling pathway. Shen et al. demonstrated in a murine model of spontaneous mammary carcinoma that Notch overexpression leads to upregulation of proinflammatory cytokines, IL-1ß and CCL2, which in turn promote the recruitment of tumor-associated macrophages (29). Further, in breast cancer patients, expression data revealed a correlation between Notch activation, IL-1ß/CCL2 expression, and macrophage infiltration (29). It is possible that these findings hold true in the context of liver cancer, as NOTCH2 is amplified in 10% of HCC patients (28); however, again, further studies are needed to demonstrate a role for tumor-intrinsic Notch signaling in promoting immune escape in the context of liver cancer.

Finally, two studies have demonstrated a role for TGFß overexpression in inducing immune escape in solid malignancies. First, Mariathasan et al. showed TGFß expression from fibroblasts leads to T cell exclusion within the peritumoral stroma and subsequent resistance to anti-PD-1 immunotherapy in urothelial cancer (30). Additionally, Tauriello et al. demonstrated that stromal cell-derived TGFß overexpression induces T cell exclusion as well as prevents acquisition of Th1 effector phenotype and resistance to anti-PD-1 immunotherapy in colon cancer (31) (Figure 1). While TGFß production from stromal cells might not be considered “tumor-intrinsic,” this immune escape mechanism may be important in the context of HCC as TGFß is overexpressed in 28% of human HCCs (28). Further, TGFß has been identified in multiple HCC classification systems based on expression data from HCC patients (17, 32).

## Tumor-Intrinsic Mechanisms of Immune Escape: Other Examples in Liver Cancer

Beyond acquisition of genetic alterations that induce immune escape, there have been other tumor-intrinsic mechanisms of immune escape described in liver cancers. For example, two tumor-derived non-coding RNA molecules have been implicated in such mechanisms. Yang et al. reported that the pseudogene or long non-coding RNA RP11-424C20.2 as well as it's parental gene, UHRF1, are upregulated in HCCs and promote immune escape, in part, through the IFNgamma-mediated CTLA-4 and PD-L1 pathways (33). Similarly, Liu et al. provided a mechanism by which endoplasmic reticulum stress in HCC leads to the release of exosomes containing the microRNA miR-23a-3p, which promotes immune escape through PTEN inhibition and subsequent upregulation of PD-L1 in macrophages (34). They showed that expression of proteins related to ER-stress were positively correlated with CD68+ macrophage recruitment and PD-L1 expression in HCC tissues (34). Furthermore, co-culture of macrophages stimulated with these exosomes and T cells led to a decrease in CD8+ T cells and IL-2 production as well as an increase in apoptosis in T cells (34). Finally, they found that miR-23a-3p levels in HCCs negatively correlated with overall survival (34). Another example of tumor-intrinsic immune escape described in HCC involves epithelial-to-mesenchymal-transition (EMT). A study by Shrestha et al. investigated the association between EMT and induction of immune checkpoint expression in HCC (35). TNFalpha induced EMT in Hep3B and PLC/PRF/5 cells and led to the upregulation of PD-L1, PD-L2, CD73, and B7-H3, whereas reversal of EMT (MET) led to suppression of these markers (35). In a cohort of 422 HCC patients, they demonstrated that high expression of TNFalpha and PD-L1 is associated with poor overall survival and expression of TNFalpha and PD-L2 was associated with increased HCC recurrence (35).

Additional examples of tumor-intrinsic mechanisms of immune escape described in liver cancer involve overexpression of secreted immunomodulatory molecules. For example, Chan et al. provided a mechanism by which IL-6-activated JAK1 phosphorylates PD-L1, which then results in PD-L1 glycosylation that maintains PD-L1 stability (36). Combination of IL-6 and TIM-3 antibody blockade resulted in synergistic T cell-mediated tumor killing *in vivo* (36). Further, they identified a positive correlation between IL-6 and PD-L1 expression in HCC patients, making this a potentially relevant and targetable mechanism in HCC (36). Another study by Li et al. detected indoleamine 2,3-dioxygenase 1 (IDO1) expression in tumor cells in 109/112 HCC patients analyzed and this expression was associated with CD8+ T cell infiltration (37). They also showed that IDO1 expression is significantly correlated with IFNgamma and CD8a transcripts in HCC and this is associated with better overall as well as disease-free survival (37). Additionally, Zhu et al. demonstrated that tumor cell-intrinsic osteopontin correlates with PD-L1 expression and tumor-associated macrophage infiltration in tumor tissues from HCC patients (38). Mechanistically, they showed that oseopontin promotes chemotactic migration of macrophages and PD-L1 expression in HCC through activation of CSF1R pathway (38). *In vivo*, dual blockage of PD-L1 and CSF1R resulted in enhanced anti-tumor immune responses and resulted in improved survival in mice with high expression of osteopontin (38). This was attributed to increased CD8+ T cell infiltration, reduced tumor-associated macrophages, as well as polarization of Th1 responses (38).

In addition to tumor-derived secreted molecules, overexpression of other molecules as well as surface receptors on tumor cells have been implicated in promoting immune escape in liver cancer. For example, Qiu et al. suggested a role for Annexin A2 in promoting immune escape in HCC by leading to an increase in regulatory T cells and expression of inhibitory molecules as well as a decrease in natural killer cells and dendritic cells (39). In another study, Zhou et al. demonstrated that tumor cell-intrinsic TLR9 activation negatively regulates PARP1 expression, promoting STAT3 phosphorylation, and leading to increased transcription of PD-L1 (40). They also show that TLR9 is positively correlated with increased STAT3 phosphorylation and PD-L1 expression while negatively associated with PARP1 expression in HCC patients (40). Finally, they demonstrated that combination therapy with TLR9 agonist and anti-PD-1 or anti-PD-L1 therapy inhibited HCC growth *in vivo* (40). Another example involved overexpression of decoy receptor 3 (DcR3) in HCC mediated by the TGFß-Smad-Sp1 pathway. Overexpression of DcR3 promotes Th2 and regulatory T cell while inhibiting Th1 differentiation and knockdown of DcR3 restored CD4+ T cell immunity (41). Another study by Ren et al. provided a mechanism by which CD147 expression on HCC tumor cells promotes immune escape through binding secreted cyclophilin A (42). This subsequently led to tumor cell proliferation through ERK1/2 pathway activation and knockdown of CD147 Hepa1-6 cells led to increased T cell chemotaxis (42).

Other studies have also demonstrated expression of immune checkpoint inhibitors on liver cancer cells, which attenuate anti-tumor immune responses. In this regard, Li et al. defined 5 subtypes of stage I/II HCCs based on gene expression profiles from TCGA, gene expression omnibus, and the International Cancer Genome Consortium that each differ in immune profile and clinical responses (43). For example, subtype C4 was associated with upregulation of immune profiles as well as expression of immune checkpoint inhibitors (e.g., PDCD1, CD274, CTLA4, etc.) whereas subtype C5 was associated with downregulation of the same immune profiles (43). Similarly, Zhou et al. characterized tumor-infiltrating lymphocytes from HCC patients who underwent surgical resection and found higher expression of PD-1, TIM3, LAG3, and CTLA4 on CD8+ and CD4+ T cells isolated from tumor tissue compared with control tissue or blood (44). They also found expression of PD-1, TIM3, and LAG3 was higher on tumor-specific CD8+ T cells compared with other CD8+ T cells (44). Tumor infiltrating lymphocytes with expression of these checkpoint inhibitors had higher expression of activation markers, but similar or lower levels of granzyme B expression compared to tumor infiltrating lymphocytes not expressing these checkpoints (44). Blocking antibodies against these checkpoints resulted in increased proliferation of CD8+ and CD4+ tumor infiltrating lymphocytes and cytokine production in response to stimulation (44). Another interesting study by Li et al. set out to investigate the mechanism of resistance of HCCs to MET inhibitors (45). They found that MET inhibitors promote immune escape through stabilization of PD-L1 and decreased anti-tumor T cell inactivation (45).

## Mechanisms of Immune Escape: Immune Infiltrates in Liver Cancer

In addition to tumor-intrinsic mechanisms of immune escape, some studies in liver cancer have described immune escape due to perturbations within the tumor-immune microenvironment. First, Dong *et al*. demonstrated in a cohort of 15 patients with multifocal HCC^+^ that those arising from intrahepatic metastasis vs. multicentric occurrence had a unique tumor-immune microenvironment (46). Specifically, those with multicentric occurrence had higher expression of immune checkpoint inhibitors and higher levels of immunoediting while those with intrahepatic metastasis had less T cell and M2-like macrophage infiltration (46). Another study found an association between M1-like macrophage infiltration and PD-L1 expression in HCC and further demonstrated that M1-conditioned media from THP-1 cells induced expression of PD-L1 in HCC cells (47). In this study, they identified IL-1ß to be the major driver of PD-L1 expression through transcription factors p65 and IRF1 (47). Additionally, Liu et al. found a role for CCL15-mediated recruitment of CCR1+CD14+ monocytes in promoting tumor invasion and metastasis and these tumor-derived monocytes also expressed high levels of immunosuppressive molecules including PD-L1, B7-H3, and TIM3 (48). Moreover, CCR1+CD14+ monocytes positively correlated with CCL15 expression and predicted survival in HCC patients (48). Aside from macrophages and monocytes, Ye et al. found that HCC patients show higher TIM-1+ regulatory B cell infiltration within tumors compared to peri-tumoral sites, and that these cells express IL-10 and promote CD8+ T cell suppression (49). Mechanistically, this was shown to be due to HMGB1 from tumor-derived exosomes, which lead to activation of B cells and expansion of TIM-1+ regulatory B cells through TLR2/4 and MAPK pathways (49). The accumulation of TIM-1+ regulatory B cells was associated with advanced stage HCC and was associated with reduced survival and predicted early recurrence of disease (49). Another interesting study by Kang et al. compared conventional HCCs (cHCCs) with HCCs containing immune cell stroma (isHCCs) and found that isHCCs had higher Epstein-Barr virus (EBV)-positivity in CD20+ tumor infiltrating lymphocytes (50). isHCCs also had higher CD8+ T cell infiltration, PD-L1 and PD-1 expression in tumor infiltrating lymphocytes, PD-L1 expression in tumors, and association with a favorable recurrence-free survival (50). However, paradoxically, a subgroup of isHCCs with high EBV-positivity in tumor-infiltrating lymphocytes demonstrated poorer recurrence free and overall survival as well as higher enrichment scores for CD8+ T cell exhaustion (50). Furthermore, *CTNNB1* mutations were not identified in isHCCs, whereas 24.1% of cHCCs harbored such mutations (50). Interestingly, viral infections such as hepatitis B precede many cases of liver cancer and these viral infections can lead to expression of unique viral antigens. However, there are also tumor antigens that are produced due to mutations generated throughout the process of tumorigenesis. A study conducted by Bubie et al. provided strong evidence that tumor neoepitopes are more immunogenic than viral epitopes in hepatitis B virally infected liver cancer and that this could potentially drive immune response in this context (51).

## Conclusions

As an immune privileged site, the liver can tolerate the introduction of innocuous antigens without mounting an immune response (52). This is necessary as the hepatic portal system brings blood through the portal vein and hepatic arteries. The portal vasculature supplies blood from the gastrointestinal tract, spleen, and associated organs whereas the hepatic arteries bring oxygenated blood from the aorta. Though immune privileged, the liver is enriched in immune cells. The liver has the largest reservoir in the body of tissue-resident macrophages, which are called Kupffer cells (53). Additionally, the liver contains resident γδT cells, natural killer cells, B cells, and other antigen presenting cells (54, 55). More comprehensive reviews on liver immunology have been conducted (53–55). Furthermore, underlying liver diseases (e.g., hepatitis viral infections, alcohol abuse, or non-alcoholic fatty liver disease) occur in the majority of patients with HCCs (1), meaning most cases of HCCs arise in the context of chronic inflammation. Thus, tumor immunology in the context of liver cancer is likely a critical factor in disease initiation and progression. This is further supported by the recent unprecedented success of immunotherapies in the treatment of advanced HCC. However, there is still very little known regarding tumor-intrinsic mechanisms of modulating immune responses specifically in the context of liver cancer, but also in most tumor types in general. As a very heterogeneous disease, this is an exciting area of study in HCC and lends the opportunity to design personalized combination immunotherapies for patients with advanced HCC that are rationally designed based on unique genetic alterations and the mechanisms by which these genetic alterations induce immune escape. However, much more mechanistic work in this regard needs to be conducted.

## Author Contributions

KL drafted the manuscript. KL, MR, and AL reviewed and revised the manuscript. KL and AL conceived the review. All authors contributed to the article and approved the submitted version.

## Conflict of Interest

Research grants from Pfizer and Genentech for unrelated projects AL. The remaining authors declare that the research was conducted in the absence of any commercial or financial relationships that could be construed as a potential conflict of interest.
